# A Mobile Intervention Designed Specifically for Older Adults With Frailty to Support Healthy Eating: Pilot Randomized Controlled Trial

**DOI:** 10.2196/50870

**Published:** 2023-11-15

**Authors:** Yan Su, Kuan-Ching Wu, Shao-Yun Chien, Aishwarya Naik, Oleg Zaslavsky

**Affiliations:** 1 College of Nursing & Health Sciences University of Massachusetts Dartmouth Darmouth, MA United States; 2 School of Nursing University of Washington Seattle, WA United States; 3 Human Centered Design and Engeneering University of Washington Seattle, WA United States

**Keywords:** frailty, healthy eating, mobile, mHealth, mobile health, app, apps, clinical trial, Mediterranean diet, metabolic health, diet, dietary, RCT, randomized controlled trial, randomized, controlled trial, controlled trials, frail, eating, food, nutrition, adherence

## Abstract

**Background:**

Frailty, a common geriatric syndrome, predisposes older adults to functional decline. No medications can alter frailty's trajectory, but nutritional interventions may aid in supporting independence.

**Objective:**

This paper presents a pilot randomized controlled trial to investigate the feasibility and efficacy of a mobile health intervention, “Olitor,” designed to enhance adherence to the Mediterranean diet among older adults with frailty, requiring no external assistance.

**Methods:**

The study sample consisted of 15 participants aged 66-77 (mean 70.5, SD 3.96) years randomized into intervention (n=8; 8 females; mean 72.4, SD 4.8 years) and control groups (n=7; 6 females, 1 male; mean 70.0, SD 3.9 years). The intervention involved a patient-facing mobile app called “Olitor” and a secure web-based administrative dashboard. Participants were instructed to use the app at least weekly for 3 months, which provided feedback on their food choices, personalized recipe recommendations, and an in-app messaging feature. Using Mann-Whitney tests to compare change scores and Hedges *g* statistics to estimate effect sizes, the primary efficacy outcomes were adherence to the Mediterranean diet score and insulin resistance measures. Secondary outcomes included retention as a measure of feasibility, engagement level and user app quality ratings for acceptability, and additional metrics to evaluate efficacy. Models were adjusted for multiple comparisons.

**Results:**

The findings demonstrated a significant improvement in the Mediterranean diet adherence score in the intervention group compared to the control (W=50.5; adjusted *P*=.04) with median change scores of 2 (IQR 2-4.25) and 0 (IQR –0.50 to 0.50), respectively. There was a small and insignificant reduction in homeostasis model assessment of insulin resistance measure (W=23; adjusted *P*=.85). Additionally, there were significant increases in legume intake (W=54; adjusted *P*<.01). The intervention's effect size was large for several outcomes, such as Mediterranean diet adherence (Hedges *g*=1.58; 95% CI 0.34-2.67) and vegetable intake (Hedges *g*=1.14; 95% CI 0.08-2.21). The retention rate was 100%. The app's overall quality rating was favorable with an average interaction time of 12 minutes weekly.

**Conclusions:**

This pilot study revealed the potential of the mobile intervention “Olitor” in promoting healthier eating habits among older adults with frailty. It demonstrated high retention rates, significant improvement in adherence to the Mediterranean diet, and increased intake of recommended foods. Insulin resistance showed a minor nonsignificant improvement. Several secondary outcomes, such as lower extremity function and Mediterranean diet knowledge, had a large effect size. Although the app's behavior change features were similar to those of previous digital interventions, the distinctive focus on theory-informed mechanistic measures involved in behavioral change, such as self-regulation, self-efficacy, and expected negative outcomes, may have enhanced its potential. Further investigations in a more diverse and representative population, focusing on individuals with impaired insulin sensitivity, are warranted to validate these preliminary findings.

**Trial Registration:**

ClinicalTrials.gov NCT05236712; https://clinicaltrials.gov/study/NCT05236712

## Introduction

Frailty is a substantial, common, and potentially modifiable geriatric syndrome heightening vulnerability to functional decline [1]. Currently, according to clinical guidelines for managing frailty, no medications can change frailty's trajectory, but nutritional interventions may help preserve function in affected individuals [2]. Approaches for enhanced nutrition include nutrient supplements, which have shown mixed yet positive effects on physical function in frail individuals [3]. Recent dietary guidelines advocate for a comprehensive healthy diet rather than focusing on individual foods and nutrients to foster sustained health behavior changes [4]. The Mediterranean diet, which places a strong emphasis on consuming ample amounts of fruits, vegetables, legumes, whole grains, olive oil, herbs, and nuts, a moderate intake of red wine and prioritizes fish and seafood over animal protein [5], has been reported as reducing incident frailty with robust evidence [6]. Mediterranean diet may affect frailty through its links to cardiovascular health, inflammation, and glucose metabolism [7-9]. The latter is particularly relevant since disruptions in glucose metabolism seem to be connected to frailty and functional loss [10].

Although self-management is appropriate for dietary interventions, personalized approaches are underused in older populations [11]. Modern mobile technologies, which are pervasive, advanced, and increasingly popular among older individuals in the United States, offer the potential to enable tailored self-management interventions [12]. Recently, mobile health (mHealth) interventions have emerged to support the self-management of various chronic health conditions [13,14]. Numerous health-promoting mobile interventions have been developed, including those promoting healthier diets [15,16]. However, most interventions are not designed for older frail users, lack theory-based strategies, and have not been evaluated in randomized trials [17]. Many older individuals may have lower digital literacy compared to younger populations, and systems repurposed for older users often overlook the vital characteristics and usability needs of this demographic [18]. Insufficient theorization of dietary behavior interventions is still common [19], despite national calls for increased focus on reproducible mechanisms of change, including specifying change mechanisms and applying behavior change theory [20]. To bridge these gaps, we conducted a pilot randomized controlled trial (RCT) to assess the feasibility and preliminary efficacy of an innovative, theory-driven mobile intervention specifically designed for older adults with frailty, aimed at increasing Mediterranean food consumption [21]. Feasibility was determined by the number of participants enrolled and retained. Primary efficacy outcomes included adherence to the Mediterranean diet score and insulin resistance measures. Secondary and theoretical mechanistic variables involved in behavioral change were food groups, social cognitive theory constructs, and anthropometric and functional measures [22,23]. Usability and mobile app analytics provided deeper insights into participants' experiences and engagement with the app identifying potential areas for improvement [24].

## Methods

### Overview

**Figure 1:**
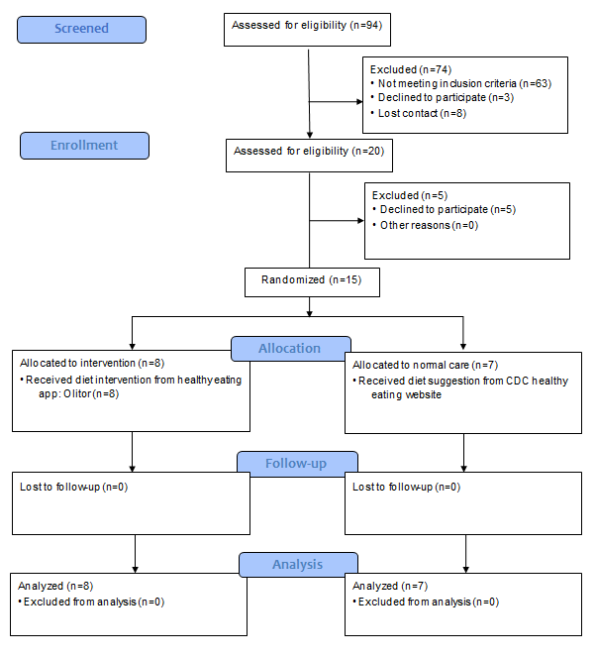
CONSORT (Consolidated Standards of Reporting Trials) flow diagram of participant progression through the phases of the pilot randomized controlled trial comparing intervention and control groups. CDC: Centers for Disease Control and Prevention.

We adhered to the CONSORT (Consolidated Standards of Reporting Trials) 2010 updated guidelines for designing and reporting the parallel-group randomized trial [25] (Figure 1; Multimedia Appendix 1).

### Ethical Considerations

The study protocol was approved by the institutional review board at the University of Washington (STUDY00012842) on April 26, 2021.

### Study Design

This was a pilot RCT to test the feasibility and preliminary efficacy of a mobile intervention consisting of a patient-facing mobile app and a secure web-based administrative dashboard. Participants were randomly assigned to either the intervention group or the control group. The process of simple random sampling was executed by one of the coauthors, a principal investigator not involved in human-subject interactions. This was carried out using a web-based random number generator [26], a tool that allows users to arrange list elements into a random sequence. Those randomized to the intervention received access to a mobile app, available for download by invitation only on the Apple App Store. Participants in the control group were emailed a referral to access materials from the National Institute on Aging regarding healthy eating [27].

### Power Calculations

In this pilot study, power calculations were calibrated to determine the effect sizes of the intervention. Referring to the MedD intervention study by Rallidis et al [28] and focusing on insulin resistance primary outcome, a sample size of 162 was determined using an 80% power threshold with an α of .05 (2-tailed). Following the conventional recommendation, which proposes 9% of the main trial's expected sample size [29] for a pilot RCT, 15 participant sample size was targeted.

### Participants

Study participants were recruited between January and October 2022 through listserve emails, presentations, and flyers in retirement communities and other places frequented by older adults in Seattle, Washington. Eligibility criteria included (1) being age 65 years and older and (2) self-reported having difficulty with at least one of the following activities suggestive of frailty: performing heavy housework, performing light housework, walking outside alone, managing money or paying bills, shopping for personal items, and preparing meals. The frailty screening was based on core components from a previously validated self-report measure [30,31]. As we aimed to recruit participants exhibiting some level of frailty, identifying any deficits was deemed an indication of their eligibility for the trial. Exclusion criteria included (1) having difficulty with basic activities of daily living such as eating and getting out of bed, suggestive of overt disability; (2) having a Memory Impairment Screen for Telephone score of <3, suggestive of memory impairment or cognitive difficulties; (3) having a 14-item Mediterranean Diet Adherence Screener >8, suggestive of an optimal diet; and (4) severe hearing or visual impairment. Eligible participants were compensated for a total of US $250. A sample size of 15 was determined based on power calculations for a pilot RCT [29].

### Intervention Components

#### Patient-Facing Mobile App

##### Overview

The app’s name “Olitor,” designed to improve adherence to the Mediterranean diet, is derived from 2 components. The first part, “Oli-,” comes from the word “olives,” which are widely recognized as a key feature of the Mediterranean diet. The second part, “-tor,” is inspired by the word “monitor,” emphasizing the app's role in tracking and supporting users' dietary experience. Combining these elements, “Olitor” reflects the app's purpose in promoting the consumption of Mediterranean foods, particularly those rich in healthy fats such as olives, while monitoring users' progress toward healthier eating habits. The app was developed by researchers from the University of Washington School of Nursing using iterative human-centered design methods [32] with strong involvement of the target population. The corresponding author is the developer of the app. The behavioral change features within the app were developed based on the social cognitive theory, a common theoretical framework used in dietary interventions [22]. Specifically, the app allows users to track their meals, receive feedback on their food choices, receive personalized recommendations for healthy recipes, and ask questions via chat.

##### Self-Tracking and Immediate Feedback

Once the app is activated, individuals are prompted to engage with the system at least weekly by receiving automatic reminders. Pressing the reminder launches a short series of questions with multiple-choice response options focusing on recent meals. The questions are modeled after the 14-item Mediterranean Adherence Screener that has demonstrated convergent validity with the food frequency questionnaire (FFQ) alternate Mediterranean Diet Index [33]. The question interfaces were designed to be aesthetically pleasing and intuitive enough to support recurrent self-tracking. Based on responses to the survey, the app provides an intuitive visualization of the survey results and personalized suggestions on improving the person’s diet. Specifically, the system displays the ideal intake levels for recommended foods and the user's current consumption levels. Should any discrepancies arise, the system offers strategies to reach the desired intake levels.

##### On-Demand Resources

In addition to responding to the survey, individuals can access on-demand resources anytime, including an information page about the Mediterranean diet and personalized recipes for breakfast, lunch, dinner, and snacks generated using a proprietary machine learning algorithm. The recipe page includes step-by-step instructions on how to prepare Mediterranean foods along with a list of ingredients needed to prepare the meal. The recipes are tailored based on survey results, bridging the gap between recommended intake and the user's consumption. Even without identifying gaps, the system proposes recipes to sustain a nutritious diet. Furthermore, recipes are refreshed weekly to keep users engaged and provide variety.

##### In-App Messaging

Users can access notifications within the app and use a color-coded messaging system for clear communication. Tailored for older users, the digital keyboard has an enlarged font for easy readability. Clear instructions are also provided, ensuring smooth navigation and enhanced engagement for the senior demographic.

#### Web-Based Dashboard

Complementing the “Olitor” patient-facing mobile app, a secure web-based administrative dashboard was developed to support the app's functionalities and facilitate efficient monitoring and management of user engagement. The dashboard, accessible only to authorized administrators, provides an overview of user data, including meal tracking, survey responses, and in-app messaging activities. This information enables the administrators to monitor adherence to the Mediterranean diet and identify potential issues that may warrant further intervention.

The administrative dashboard offers several key features, including the following:

1. User data overview: A comprehensive summary of individual user activities, such as survey responses, suggested meals, and overall dietary scores, allows the administrators to assess overall engagement and identify issues that may require attention.
2. In-app messaging support: The dashboard facilitates efficient monitoring and management of in-app messaging interactions, enabling the research team to respond promptly to user inquiries and provide additional guidance as needed.

### Data Collection Procedures

A study coordinator collected data during in-person assessments and remotely using standardized procedures at 2 time points. At the baseline in-person assessment, the study coordinator administered clinical and demographic questionnaires, collected functional and anthropometric measures, and conducted a blood draw using dried blood spot (DBS) collection methods (see details in the Primary Measures section) [34]. The second in-person visit occurred about 12 weeks later and included a similar battery of assessments and a recording of a semistructured exit interview. Moreover, to better understand user engagement and interaction with the mHealth intervention, we collected and analyzed user log data. User log data were automatically captured and stored by the app's backend system, ensuring an accurate and comprehensive collection of user interactions. These data comprised a record of users' actions within the app, including the frequency and duration of use, features accessed, and progress through various intervention components.

### Measures

Baseline questionnaires included the following demographic variables: self-reported age, sex, educational attainment, race, and ethnicity**.**

#### Primary Measures

The primary measures are as follows: (1) MedD adherence was measured using an FFQ and adherence to MedD (aMED) score [35,36]. The self-administered FFQ asks participants to report the frequency of consumption and portion size of approximately 125-line items over the last 3 months. A series of foods or beverages define each line item. The aMED score was designed to assess adherence to a Mediterranean dietary pattern. aMED scores range from 0 (nonadherence) to 9 (perfect adherence) [34,35]. (2) Insulin resistance was assessed using plasma glucose and insulin measures to calculate the homeostasis model (homeostasis model assessment; HOMA) [37]. Specifically, the DBS collection method was used to obtain the overnight fasting blood samples of glucose and insulin. This method was selected for its ease of use, cost-effectiveness, and minimal invasiveness compared to traditional venipuncture. DBS involves collecting small volumes of blood from a fingertip puncture, which is then spotted onto a specialized filter paper, allowed to dry, and stored for later analysis [34].

#### Secondary and Mechanistic Measures

Feasibility was determined by the number of participants enrolled and retained. Acceptability was determined by the user’s rating of the app quality and the extent and consistency of weekly interactions with the app. Other efficacy measures included individual Mediterranean food groups, behavior change mechanisms, physical performance, anthropometric, knowledge, and selected metabolic plasma measures collected using DBS. Specifically, the following aMED index components were measured: whole grains, vegetables, fruits, legumes, nuts, fish, red and processed meats, ratio of monounsaturated fat to saturated fat, and alcohol. The Food Beliefs Survey 5 subscales were used to measure behavior change mechanisms focused on MedD [38]. (1) Five items were used to assess positive outcome expectations, which involved participants rating their beliefs about the potential positive effects of increasing MedD. An example statement was “My health will improve.” Ratings were made on a 5-point scale, with 5 indicating strong agreement and 1 indicating strong disagreement [38,39]. (2) Rated on the same scale, negative outcome expectations (NOEs) were elicited with 6 items, such as a person’s expectations that, as a result of increasing MedD, “The food I eat will not taste good [37,38].” (3). Self-efficacy (SE) was measured by responses to 6 items relating to a person’s confidence that he or she will be able to increase consumption of fruits, vegetables, olive oil, nuts, whole grains, and fish using a 100-point visual analog scale [38,39]. (4). Self-regulation (SR) was measured by responses to 2 items regarding a person’s plans to keep track of servings of MedD foods they eat and intention to eat at least 3 servings of MedD foods each day using a 5-point scale (5=strongly agree and 1=strongly disagree) [38,39]. (5). Social support (SS) was measured by responses to 2 items regarding a person’s perception that other participants in the study try to adhere to MedD and try to eat at least 3 servings of MedD food each day using a 5-point scale (5=strongly agree and 1=strongly disagree) [37,38]. The Short Physical Performance Battery (SPPB) measured functional status and physical performance [40]. The scale is a composite measure assessing walking speed, standing balance, and sit-to-stand performance. A change of 0.5 points on the SPPB is a small meaningful change, while a change of 1 point on the SPPB is considered to be a substantial, meaningful change [41]. Weight to the nearest 0.1 kg and height to the nearest 0.1 cm were measured and used to compute BMI. MedD knowledge was assessed using the Mediterranean Diet Nutrition Knowledge (MDNK) questionnaire [42]. Secondary efficacy measures included metabolic analytes collected via DBS: lipids (total cholesterol, triglycerides, and high-density lipoprotein) and inflammation (C-reactive protein) [34]. Those choices of the measures were based on prior similar study [43]. The Mobile Application Rating Scale measured app quality concerning the following dimensions: engagement, functionality, aesthetics, and information [24]. Items were rated on a 5-point scale ranging from “1=inadequate” to “5=excellent.”

### Analyses

Descriptive statistics were first calculated to compare demographic characteristics between the intervention and the control groups. Outcome measures were analyzed by calculating the within-group pretest-posttest change scores and then comparing the change scores between the intervention and the control groups using Mann-Whitney tests. A *P* value smaller than .05 was considered statistically significant. Next, we used the Benjamini-Hochberg procedure to control the false discovery rate in multiple tests. This method, appropriate for pilot studies, adjusts *P* values to balance control for type I errors while retaining statistical power [44]. To estimate the magnitude of the intervention's impact compared to the control group, we used Hedges *g* as a measure of effect size. Hedges *g* is a variation of Cohen *d* that corrects for biases that may occur in small sample sizes, making it a more appropriate choice for this study. According to Lakens [45], Hedges *g* is recommended when the sample size is less than 20. Hedges *g* takes the difference in means between the intervention and control groups and divides it by the pooled SD while applying a correction factor to account for potential biases. A larger absolute value of Hedges *g* indicates a greater effect size, with values of 0.2, 0.5, and 0.8 generally considered small, medium, and large effect sizes, respectively. An R package, *BootES* was used to calculate 95% bootstrap CIs for the effect sizes [46]. The *BootES* package is designed specifically for this purpose, providing a robust and efficient method for estimating CIs based on resampling techniques. Finally, descriptive statistics and visual analytics were used to describe app quality and analyze log data of user interactions.

## Results

Table 1 summarizes the baseline data of 15 participants recruited for the trial. In total, 8 participants were randomized to the intervention, and 7 were randomized to control groups. The group's demographic characteristics were comparable such that the average age (SD) was 72.4 (4.8) years in the intervention group and 70.0 (3.9) years in the control group. The intervention group had 100% (n=8) females, and the control group had 1 male. Both groups were comparable in race and education, being predominantly White and college-educated. All participants completed baseline and postintervention measurements and consistently engaged with the study protocol, resulting in 100% retention and engagement.

Tables 2-4 summarize the pretest, posttest, and change scores for primary, secondary, and mechanistic measures between the intervention and the control groups using the Mann-Whitney test. In primary measures, there was a significant improvement in adherence to the Mediterranean diet score in the intervention compared to the control (W=50.5; adjusted *P*=.04), such that median change scores were 2 (IQR 2-4.25) and 0 (IQR –0.50 to 0.50), respectively. There was also a small but insignificant improvement in insulin resistance measures in the intervention compared to the control (W=23; adjusted *P*=.85), such that the mean changes were –0.18 (SD 0.72) and 0.10 (SD 0.74), respectively. Another significant improvement was observed in legumes (W=54; adjusted *P*<.01). Although not reaching the level of conventional statistical significance, all other measures trended in the expected direction. For example, participants showed an increased intake of vegetables (W=47; adjusted *P*=.08), improvement in knowledge about the Mediterranean diet (W=49.5; adjusted *P*=.1), and improvement in SPPB measure (W=45; adjusted *P*=.13).

**Table 1:** Demographic characteristics for both the intervention and control groups.

Variable		Intervention (n=8)	Control (n=7)
Age (years), mean (SD)		72.4 (4.8)	70.0 (3.9)
**Gender, n (%)**			
	Female	8 (100)	6 (86)
	Male	0 (0)	1 (14)
**Race, n (%)**			
	White	7 (88)	7 (100)
	Asian	1 (12)	0 (0)
**Education, n (%)**			
	Associate degree	0 (0)	1 (14)
	Bachelor degree	2 (25)	2 (29)
	Master degree	4 (50)	2 (29)
	Professional degree	0 (0)	1 (14)
	Doctoral degree	0 (0)	1 (14)
	Some college, no degree	2 (25)	0 (0)
**Marital status, n (%)**			
	Never married	1 (12)	2 (29)
	Married	3 (38)	1 (14)
	Divorced	3 (38)	2 (29)
	Separated	0 (0)	1 (14)
	Widowed	1 (12)	1 (14)
Members in the household, n (%)		1.5 (0.5)	1.6 (1)

**Table 2:** Pretest scores, posttest scores, and comparison of change scores on healthy eating behavior outcomes between intervention and control groups.

		Intervention group			Control group			W		Adjusted *P* value
		Mean (SD)	Median (IQR)	Mean (SD)		Median (IQR)				
**Adherence to MedD**										
	Pretest	2.88 (2.53)	2.5 (1.5 to 3.75)	4.29 (2.29)		4 (3 to 5.50)	N/A^a^		N/A	
	Posttest	5.5 (2)	5 (5 to 6.25)	4 (1.73)		3 (3 to 5)	N/A		N/A	
	Change	2.63 (2.0)	2 (2 to 4.25)	–0.29 (1.38)		0 (–0.50 to 0.50)	50.5		.04	
**Vegetables**										
	Pretest	2.46 (0.73)	2.32 (1.87 to 2.89)	3.37 (1.11)		3.22 (2.58 to 3.98)	N/A		N/A	
	Posttest	3.22 (1.68)	3.18 (1.98 to 4.33)	2.62 (0.88)		2.45 (2.11 to 3.02)	N/A		N/A	
	Change	0.76 (1.28)	0.70 (0.20 to 1.58)	–0.75 (1.20)		–0.95 (–1.38 to 0.28)	47		.08	
**Nuts**										
	Pretest	1.46 (1.38)	0.87 (0.60 to 2.02)	2.70 (1.11)		2.458 (2.20 to 3.34)	N/A		N/A	
	Posttest	1.94 (2.14)	1.26 (0.35 to 2.47)	2.2972 (2.11)		2.23 (1.21 to 2.36)	N/A		N/A	
	Change	0.47 (2.23)	–0.23 (–0.35 to 0.76)	–0.40 (2.22)		–0.97 ( –1.58 to –0.39)	41		.20	
**Whole grains**										
	Pretest	1.99 (2.09)	1.25 (0.96 to 2.18)	2.02 (1.46)		1.36 (1.21 to 2.58)	N/A		N/A	
	Posttest	2.71 (1.57)	3.01 (1.67 to 3.96)	1.81 (1.60)		1.33 (0.74 to 2.80)	N/A		N/A	
	Change	0.72 (1.44)	1.07 (0.20 to 1.67)	–0.21 (0.78)		–0.30 (–0.68 to 0.38)	42		.19	
**Fruits**										
	Pretest	1.11 (0.63)	0.94 (0.64 to 1.76)	1.371 (0.93)		1.31 (0.74 to 1.92)	N/A		N/A	
	Posttest	1.22 (0.96)	0.79 (0.53 to 1.93)	1.41 (0.95)		1.8 (0.73 to 1.98)	N/A		N/A	
	Change	0.11 (0.82)	0.17 (–0.19 to 0.47)	0.04 (0.44)		0.18 (–0.16 to 0.27)	29		.96	
**Fish**										
	Pretest	1.482 (1.34)	1.20 (1.06 to 1.36)	1.117 (0.83)		0.75 (0.67 to 1.60)	N/A		N/A	
	Posttest	1.98 (1.37)	1.69 (1.45 to 2.41)	0.76 (0.95)		0.49 (0.13 to 0.92)	N/A		N/A	
	Change	0.50 (0.73)	0.30 (–0.01 to 0.73)	–0.36 (0.84)		–0.65 (–0.67 to 0.28)	42		.19	
**Legumes**										
	Pretest	0.09 (0.11)	0.04 (0.01 to 0.15)	0.24 (0.16)		0.13 (0.13 to 0.31)	N/A		N/A	
	Posttest	0.17 (0.16)	0.25 (0.04 to 0.26)	0.14 (0.09)		0.152 (0.08 to 0.21)	N/A		N/A	
	Change	0.08 (0.13)	0.03 (0.00 to 0.13)	–0.10 (0.09)		–0.07 ( –0.13 to –0.04)	54		<.001	
**Meat**										
	Pretest	1.38 (1.11)	1.64 (0.35 to 1.97)	1.10 (0.69)		0.52 (0.68 to 1.53)	N/A		N/A	
	Posttest	0.69 (0.59)	1.39 (0.27 to 0.91)	0.90 (1.22)		0.46 (0.07 to 1.12)	N/A		N/A	
	Change	–0.69 (0.79)	–0.46 (–1.38 to –0.05)	–0.19 (0.96)		–0.23 (–0.72 to 0.06)	21		.51	

^a^N/A: not applicable.

**Table 3:** Pretest scores, posttest scores, and comparison of change scores on metabolic outcomes between intervention and control groups.

		Intervention group			Control group			W		*P* value
		Mean (SD)	Median (IQR)	Mean (SD)		Median (IQR)				
**Total cholesterol**										
	Pretest	140.75 (35.78)	139 (111.5 to 164.2)	112.57 (32.49)		113 (90.0 to 137.0)	N/A^a^		N/A	
	Posttest	127.13 (34.11)	133.5 (106.0 to 145.8)	116.71 (32.27)		122 (89.5 to 143.0)	N/A		N/A	
	Change	–13.62 (33.04)	–15.50 (–21.50 to 3.75)	4.14 (15.55)		6.00 (–8.50 to 10.00)	16		.85	
**C-reactive protein^b^**										
	Pretest	3.31 (4.46)	1.15 (0.66 to 4.10)	2.72 (0.96)		2.7 (2.58 to 3.04)	N/A		N/A	
	Posttest	2.07 (2.43)	1.17 (0.86 to 1.94)	2.45 (1.14)		2.59 (1.75 to 3.44)	N/A		N/A	
	Change	–1.24 (2.63)	0.03 (–2.40 to 0.33)	–0.27 (1.06)		–0.11 (–0.96 to 0.62)	21		.85	
**Plasma glucose**										
	Pretest	111.50 (25.04)	106 (95.25 to 118.75)	102.86 (12.32)		98 (95.5 to 110.5)	N/A		N/A	
	Posttest	106.62 (13.26)	104 (99.75 to 110.25)	104.57 (15.04)		98 (96.0 to 105.5)	N/A		N/A	
	Change	–4.88 (19.47)	–11.00 (–18.25 to 3.50)	1.71 (9.64)		2.00 (–2.00 to 7.50)	21.5		.85	
**High-density lipoprotein**										
	Pretest	71.25 (15.27)	72.5 (63.25 to 83.25)	60.43 (13.73)		63 (56.00 to 67.00)	N/A		N/A	
	Posttest	73.38 (21.28)	74 (66.75 to 85.75)	61.14 (9.51)		65 (53.50 to 67.50)	N/A		N/A	
	Change	2.125 (15.67)	7.50 (–11.00 to 12.00)	0.71 (7.76)		0.00 (–3.00 to 1.50)	32		.85	
**Homeostasis model assessment** **(insulin resistance score)**										
	Pretest	1.63 (1.26)	1.27 (0.71 to 2.18)	1.47 (0.50)		1.51 (1.40 to 1.77)	N/A		N/A	
	Posttest	1.46 (0.70)	1.29 (1.01 to 1.63)	1.57 (0.88)		1.23 (1.00 to 1.93)	N/A		N/A	
	Change	–0.18 (0.72)	–0.005 (–0.80 to 0.39)	0.10 (0.74)		0.03 (–0.39 to 0.59)	23		.85	
**Insulin**										
	Pretest	5.82 (4.53)	4.11 (2.68 to 7.31)	5.74 (1.95)		5.84 (5.075 to 6.975)	N/A		N/A	
	Posttest	5.57 (2.75)	4.89 (4.41 to 5.82)	5.92 (2.99)		5.08 (4.235 to 6.340)	N/A		N/A	
	Change	–0.25 (2.18)	0.81 (–2.21 to 1.09)	0.18 (2.41)		0.39 (–1.77 to 1.59)	24.5		.85	
**Triglyceride**										
	Pretest	39.63 (9.97)	40 (36.50 to 41.50)	57.71 (42.77)		43 (39.00 to 47.50)	N/A		N/A	
	Posttest	38.88 (4.32)	40.5 (35.50 to 42.00)	57.0 (34.87)		46 (40.0 to 50.5)	N/A		N/A	
	Change	–0.75 (9.71)	0 (–4.25 to 4.00)	–0.71 (10.75)		–1.00 (–5.50 to 7.00)	28		>.99	

^a^N/A: not applicable.

^b^In the analysis of C-reactive protein, one value was excluded as it was nearly 100 mg/L.

**Table 4:** Pretest scores, posttest scores, and comparison of change scores on self-report outcomes between intervention and control groups.

		Intervention group		Control group		W	*P* value
		Mean (SD)	Median (IQR)	Mean (SD)	Median (IQR)		
**BMI**							
	Pretest	26.88 (6.86)	24.25 (23.48 to 29.85)	29.04 (4.39)	28.60 (26.30 to 29.05)	N/A^a^	N/A
	Posttest	26.05 (6.84)	24.05 (22.85 to 29.12)	28.89 (3.98)	28.10 (26.80 to 28.75)	N/A	N/A
	Change	–0.825 (0.95)	–0.65 (–0.95 to –0.30)	–0.16 (0.65)	–0.10 (–0.50 to 0.00)	19	.43
**Mediterranean diet knowledge**							
	Pretest	8.5 (2.51)	9.5 (6.0 to 10.0)	11.0 (2.24)	11 (9 to 12)	N/A	N/A
	Posttest	10.50 (2.0)	11 (10.0 to 12.0)	9.86 (3.76)	11 (7.5 to 12)	N/A	N/A
	Change	2.00 (2.33)	1.50 (0.00 to 2.75)	–1.14 (1.95)	–1.0 (–1.50 to 0.00)	49.5	.10
**Negative outcome expectations**							
	Pretest	14.5 (3.59)	15 (12.75 to 16.25)	10.0 (3.51)	10 (7.5 to 11.5)	N/A	N/A
	Posttest	11.25 (2.25)	12 (10.50 to 12.25)	9.57 (2.51)	10 (8.00 to 11.00)	N/A	N/A
	Change	–3.25 (3.62)	–4.50 (–5.25 to –2.75)	–0.43 (1.90)	0.00 (–1.50 to 0.00)	9	.12
**Positive outcome expectations**							
	Pretest	17.50 (3.55)	19 (16.50 to 19.25)	20.29 (3.99)	19 (17.00 to 24.00)	N/A	N/A
	Posttest	18.75 (3.45)	19.5 (16.00 to 21.25)	21.29 (3.68)	21 (19.50 to 24.50)	N/A	N/A
	Change	1.25 (5.26)	0.50 (–1.00 to 2.50)	1.0 (3.74)	0.0 (–1.5 to 4.5)	26	.86
**Self-efficacy**							
	Pretest	38 (4.17)	37 (36 to 39.25)	46.29 (12.62)	52 (35.50 to 56.00)	N/A	N/A
	Posttest	39.75 (10.61)	40 (29.5 to 47.25)	44.29 (9.79)	43 (39.00 to 49.50)	N/A	N/A
	Change	1.75 (10.59)	–1.50 (–6.50 to 10.50)	–2.0 (10.21)	–6.0 (–9.5 to 4.0)	35	.51
**Short Physical Performance Battery**							
	Pretest	8 (1.20)	8 (7.75 to 8.25)	10 (1.69)	10 (9 to 11)	N/A	N/A
	Posttest	9.50 (1.41)	10 (8.5 to 11.0)	10.14 (1.57)	10 (9 to 11.5)	N/A	N/A
	Change	1.50 (1.20)	1.50 (0.75 to 2.25)	0.14 (0.90)	0.00 (–0.50 to 1.00)	45	.13
**Self-regulation**							
	Pretest	8.25 (1.16)	8 (8 to 9)	8.57 (2.67)	10 (8.50 to 10.0)	N/A	N/A
	Posttest	8.13 (1.36)	8 (7.75 to 8.5)	6.71 (2.56)	7 (6.00 to 8.00)	N/A	N/A
	Change	–0.125 (1.25)	0.00 (–1.00 to 0.25)	–1.86 (3.13)	–3.00 (–4.00 to –0.50)	41.5	.26
**Social support**							
	Pretest	6.38 (1.06)	6 (6 to 6.5)	7.14 (1.73)	8 (6.00 to 8.00)	N/A	N/A
	Posttest	7.88 (1.95)	8 (7.5 to 8.5)	7.0 (1.29)	6 (6.00 to 8.00)	N/A	N/A
	Change	1.5 (1.41)	2.0 (0.0 to 2.0)	–0.14 (2.19)	0.00 (–1.00 to 1.50)	40	.26

^a^N/A: not applicable.

**Figure 2:**
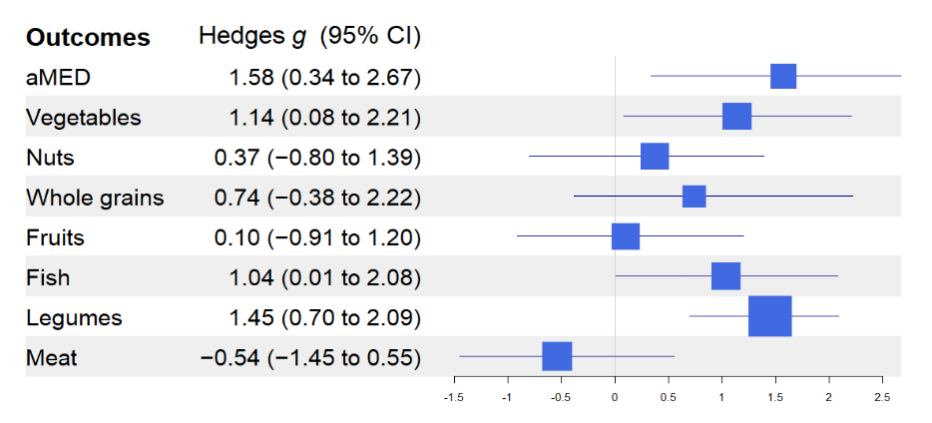
Effect sizes and CIs for healthy eating behavior outcomes. aMED: adherence to MedD.

**Figure 3:**
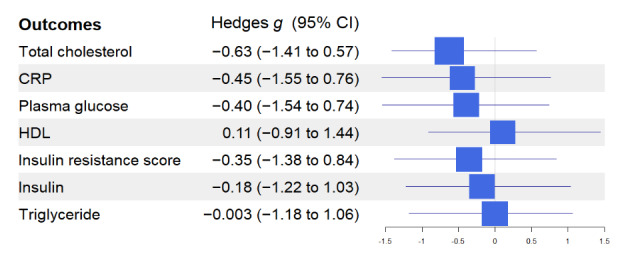
Effect sizes and CIs for metabolic outcomes. In the analysis of CRP, one value was excluded as it was nearly 100 mg/L. CRP: C-reactive protein; HDL: high-density lipoprotein.

**Figure 4:**
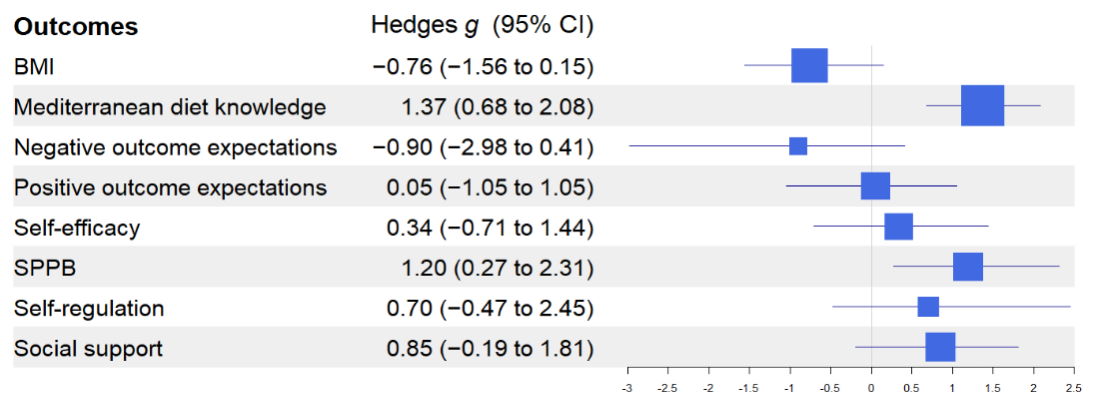
Effect sizes and CIs for self-report outcomes. SPPB: Short Physical Performance Battery.

Figures 2-4 show the effect size of the intervention using Hedges *g* statistics across all outcomes. Using described earlier heuristics concerning the magnitude of the effect size, large effect sizes were observed for the following outcomes: MDNK (Hedges *g*=1.37; 95% CI 0.68-2.08), NOE (Hedges *g*=–0.90; 95% CI –2.98 to 0.41), SPPB (Hedges *g*=1.2; 95% CI 0.27-2.31), SS (Hedges *g*=0.85; 95% CI –0.19 to 1.81), Mediterranean diet adherence (Hedges *g*=1.58; 95% CI 0.34-2.67), vegetables (Hedges *g*=1.14; 95% CI 0.08-2.21), fish (Hedges *g*=1.04; 95% CI 0.01-2.08), and legumes (Hedges *g*=1.45; 95% CI 0.70-2.09).

Table 5 shows the overall and domain-specific app quality mean (SD) rating scale results. The mean functionality score, which refers to the app's ease of learning, navigation, flow logic, and gestural design, was 4.56 (SD 0.4). The mean aesthetics score, which refers to graphic design, overall visual appeal, color scheme, and stylistic consistency, was 4.13 (SD 0.83). The mean information score, which refers to high-quality information from a credible source, was 3.77 (SD 0.59). The mean engagement score referring to the apps being fun, interesting, customizable, interactive, and well-targeted to the audience was 2.88 (SD 1.13). Finally, the app's overall mean quality score based on the 4 domains was 3.83 (SD 0.56).

**Figure 5:**
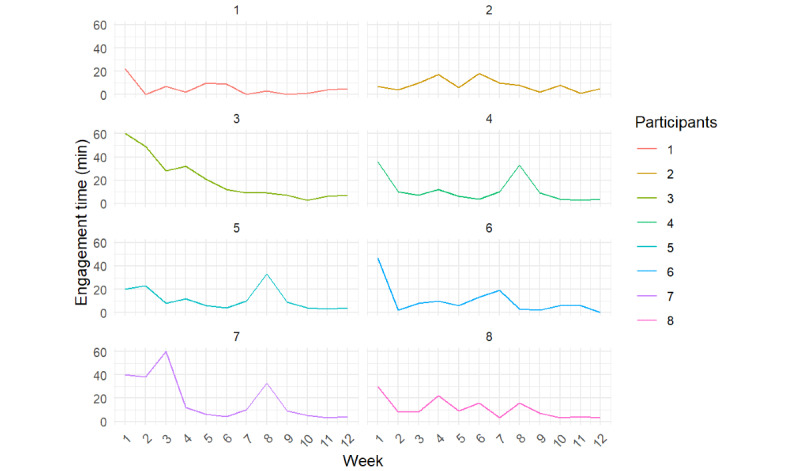
Weekly time spent interacting with the app by each participant.

Figure 5 shows individual user log data concerning the weekly time intervention group participants spent interacting with the app. The average interaction time across all participants was 12 minutes, with the shortest engagement time of 5 minutes per week and the longest of 20 minutes per week.

**Table 5:** The rating scale assesses app quality on 4 dimensions: all items are rated on a 5-point scale from “1=inadequate” to “3=acceptable” and “5=excellent” (n=8).

Four domains of items	Values, mean (SD)
Engagement: fun, interesting, customizable, interactive (eg, sends alerts, messages, reminders, and feedback and enables sharing), and well-targeted to the audience	2.88 (1.13)
Functionality: app functioning, easy to learn, navigation, flow logic, and gestural design of app	4.56 (0.4)
Aesthetics: graphic design, overall visual appeal, color scheme, and stylistic consistency	4.13 (0.83)
Information: contains high-quality information (eg, text, feedback, measures, and references) from a credible source	3.77 (0.59)
App subjective quality score	3.13 (1.44)
App quality score (engagement, functionality, aesthetics, and information)	3.83 (0.56)

## Discussion

### Principal Findings

Overall, this study showed that the theory-based mobile intervention, designed specifically for older users with frailty to support a healthier diet, was feasible and produced promising preliminary efficacy findings regarding improved overall adherence to the Mediterranean diet and increased consumption of legumes. Specifically, a 2-point increase in Mediterranean diet adherence (ie, aMED score) in the intervention and a decrease in the control arm are encouraging. Our results echo a previous well-powered observational study of 71,941 women aged 60 years and older that showed the relative risks of frailty per a 2-point increase in the aMED score were 0.87 (95% CI 0.85-0.90) [47].

Another primary measure of change in insulin resistance trended in the expected direction, reaching an effect size of –0.35 (95% CI –1.38 to 0.84) but failing to reach statistical significance. The plasma insulin score of 0.28, which compares the mean change in the intervention group to the mean change in the control group, closely mirrors the observed difference in HOMA score of 0.36 between the lowest and highest Mediterranean diet adherence tertials among National Health and Nutrition Examination Survey participants [43]. Similarly, HOMA score of 0.29 corresponds with the observed difference in insulin resistance between frail and no frailty cardiovascular health study participants [48]. Both studies showed the difference was statistically significant. Several secondary measures showed high effect sizes, including lower extremity function and Mediterranean diet knowledge. Both retention and engagement were excellent such that participants consistently engaged with the app at least once a week for, on average, 12 minutes. The app received a relatively high overall quality rating, especially concerning its functionality. Pending future research, these encouraging preliminary stage I results indicate a promising foundation for future progress according to the National Institutes of Health stage model for behavioral intervention development [49].

A strong improvement in overall adherence to the Mediterranean diet and specific foods in this study extends the latest evidence. In fact, a recent systematic review of eHealth nutritional interventions for middle-aged and older adults revealed that while eHealth interventions generally improved anthropometric and clinical outcomes, they did not consistently impact behavioral outcomes such as fruit and vegetable consumption [50]. An improved adherence in this study may be attributed to the app’s age-friendly designs, as evidenced by high scores in ease of use and the integration of behavior change frameworks. Specifically, all participants agreed that the app was easy to learn and easy to use. Furthermore, many measured behavior change constructs (a theoretical concept related to changes in behavior), such as NOE and SS, trended in the expected directions with large effect sizes. Worth noting that although many of our app’s behavior change features, such as shaping knowledge, goal setting, feedback, and reminders, were similar to most previous eHealth interventions that did not show a change in eating behaviors [38], our innovation lies in that several other behavior change techniques (SR, SE, and expected negative outcomes) were in concordance with and explicitly informed by the social cognitive theory framework. Our previous work suggested that such concordance relates to improved retention and engagement rates in nutrition interventions [19].

Insulin resistance measures showed a small, insignificant improvement. These findings are at odds with recent evidence. Specifically, a recent study showed that an 8-week Mediterranean diet intervention reversed insulin resistance among obese people without diabetes [51]. Another study showed that a 12-week Mediterranean diet intervention reversed insulin resistance among people diagnosed with diabetes [52]. One of the potential reasons for the underwhelming results in this project concerning metabolic changes is that the inclusion criteria focused on participants' functional rather than metabolic health. In fact, many of the study participants had relatively unimpaired glucose and insulin measures at baseline. Another reason might relate to the relatively short duration of the intervention. Indeed, according to a large longitudinal study among healthy older women, a longer time is needed for the Mediterranean diet to exert its benefits on insulin resistance and diabetes occurrence [53]. As such, the results must be replicated in a study that purposefully samples older adults with metabolic deficiencies and considers a longer duration of the intervention.

Previous research confirmed that better adherence to the Mediterranean diet is associated with higher physical function [53-58]. These findings have been observed in both Mediterranean and non-Mediterranean countries, spanning a period of up to 13 years among older adults [53-58]. While the exact mechanisms linking the Mediterranean diet to reduced physical function decline remain unclear, various studies underscore its potential benefits, such as anti-inflammatory and antioxidant effects, metabolic improvements, and mitigation of mitochondrial dysfunction and stem cell exhaustion, which may collectively slow age-related physical dysfunction [58]. This study supports and further extends these findings by showing an effect size of 1.2, highlighting the benefits of the Mediterranean diet in persons with frailty. Specifically, our research revealed that positive changes in both diet and lower extremity function were observed in the intervention arm. It is possible that the functional improvement can be partially attributed to alterations in diet. To date, the majority of longitudinal studies investigating the Mediterranean diet and its impact on physical function have relied on observational approaches that assume dietary stability over time. However, these studies consistently highlight the necessity of collecting longitudinal dietary data rather than assuming a constant dietary pattern. In alignment with these findings, this study emphasizes the importance of collecting longitudinal dietary habits as opposed to assuming stability over time. This approach enables us to delve into the influence of the Mediterranean diet on both the initial physical function levels and the rate of decline over time among older adults [59]. Although we have to acknowledge that low extremity performance cannot represent frailty, the SPPB score seemed a useful measure for identifying the physical frailty phenotype [60].

We observed a substantial effect size of 1.37 in Mediterranean diet knowledge. This finding holds practical significance, as previous studies have emphasized the crucial role of improved knowledge in promoting adherence to the Mediterranean diet [61]. This finding aligns with the frequent use of shaping knowledge as a behavior change technique in interventions aimed at promoting healthy eating among older adults [19]. Because the MDNK questionnaire included items pertaining to benefits and suitable substitutions [42], this knowledge might empower individuals by deepening their understanding of associated trade-offs, leading to informed choices and finding suitable substitutions, and making practical adjustments for long-term adherence. In fact, concurrent improvement in knowledge and mechanistic measures involved in behavioral change suggest opportunities for sustainable healthy eating practices. Besides nutrition knowledge, individuals' psychological measures such as SE, SR, and SS showed positive trends. These findings align with the principles of social cognitive theory, which emphasizes the importance of enhancing SE through education, training, and goal setting [22]. Our findings indicated that interventions that explicitly integrate theory and include behavior change techniques that activate corresponding mechanistic features have the potential to help individuals adopt and sustain adherence to the Mediterranean diet.

### Limitations

The study has several limitations. First, the sample was relatively skewed toward higher education and lower gender diversity, which is consistent with other eHealth studies in the comparable population. As such, the findings may not apply to be broadly generalizable. Indeed, a previous study showed that the effect of mHealth intervention on healthy eating patterns is gender-specific, with the improvement in sugar-sweetened beverages only appearing in women [62]. Another limitation is insufficient statistical power for our analyses. As such, insignificant findings do not necessarily mean the absence of an intervention effect. In pilot studies, researchers recommended prioritizing descriptive statistics and estimation using 85% or even 75% CIs over formal hypothesis testing with 95% CIs [63]. By adhering to this recommendation, it is plausible that nonsignificant findings in this study may become significant. Therefore, in addition to absolute change scores, we also provided, as a reference, effect size estimates to assist with interpreting the findings. Next, the mHealth trial carries inherent limitations, such as the impossibility of blinding, leading participants to experience anticipated positive effects from using the intervention. Moreover, mHealth trials typically involve multiple outcomes, increasing the risk of type I errors. Although we adjusted the *P* values for Mann-Whitney *U* tests and despite the fact that effect size estimation is not affected by the multiple comparisons, our CIs were not correspondingly adjusted, given the unconventional application of the Benjamini-Hochberg method to CI adjustments. Next, although we recommended that the study participants keep their habitual levels of physical activity, we did not monitor such changes and cannot rule out their confounding effects. Finally, by providing compensation, there was a potential that participants would be motivated to remain engaged in the study, which could account for our observed 100% retention rate. Our previous research has shown that offering material compensation is associated with higher retention rates in healthy eating interventions among older adults [19].

### Recommendations for Future Research

The promising preliminary results indicate a strong foundation for programmatic developments according to the National Institutes of Health stage model [49]. The intervention will benefit from larger well-powered RCTs in a more diverse population. The future trial will need to account for the confounding effects of physical activity. A previous study showed that many eHealth interventions adopted a multiple-component approach [50], especially concerning physical activity [64]. Yet, whether such an approach is synergetic or counterproductive regarding behavioral change is unclear. Our recent study showed that “less is more,” and more behavioral change elements packed into a digital intervention might lead to a lower engagement [65]. Finally, the app might further benefit from improvements, especially in the engagement domain, because only 50% (n=4) of participants partly or completely agree that the app is fun, interesting, and interactive.

### Conclusions

The study's results showed that the Olitor app has the potential to promote healthy eating among older adults with frailty. We observed consistently high retention rates and significant overall improvement in adherence to the Mediterranean diet and its recommended foods. Furthermore, the app was user-friendly and had encouraging results concerning changes in knowledge about the Mediterranean diet and lower extremity physical function. We also noted positive trends in various anthropometric measures and behavior change mechanisms. These promising initial findings suggest an opportunity for stage II work.

## Appendix

Multimedia Appendix 1 CONSORT (Consolidated Standards of Reporting Trials) eHealth checklist (V 1.6.1).

## Abbreviations

aMED: adherence to MedD
CONSORT: Consolidated Standards of Reporting Trials
DBS: dried blood spot
FFQ: food frequency questionnaire
HOMA: homeostasis model assessment
MDNK: Mediterranean Diet Nutrition Knowledge
mHealth: mobile health
NOE: negative outcome expectation
RCT: randomized controlled trial
SE: self-efficacy
SPPB: Short Physical Performance Battery
SR: self-regulation
SS: social support
